# Friend Turns Foe: Transformation of Anti-Inflammatory HDL to Proinflammatory HDL during Acute-Phase Response

**DOI:** 10.1155/2011/274629

**Published:** 2010-11-25

**Authors:** Hima Bindu G, Veena S. Rao, Vijay V. Kakkar

**Affiliations:** ^1^Tata Proteomics and Coagulation Unit, Thrombosis Research Institute, Bangalore, India; ^2^Narayana Hrudayalaya Hospital, Bangalore, India; ^3^Thrombosis Research Institute, London, UK

## Abstract

High-density lipoprotein (HDL) is a major carrier of cholesterol in the blood. Unlike other lipoproteins, physiological functions of HDL influence the cardiovascular system in favorable ways except when HDL is modified pathologically. The cardioprotective mechanism of HDL is mainly based on reverse cholesterol transport, but there has been an emerging interest in the anti-inflammatory and antioxidant roles of HDL. These latter activities of HDL are compromised in many pathological states associated with inflammation. Further, abnormal HDL can become proinflammatory contributing to oxidative damage. In this paper, we discuss the functional heterogeneity of HDL, how alterations in these particles in inflammatory states result in loss of both antioxidant activity and reverse cholesterol transport in relation to atherosclerosis, and the need for assays to predict its functionality.

## 1. Introduction

High-density lipoprotein (HDL) is a plasma lipoprotein heterogeneous in origin, size, composition, and function. It is a major carrier of cholesterol in blood, and unlike other lipoproteins, physiological functions of HDL influence the cardiovascular system favorably unless it is modified pathologically. The atheroprotective role of HDL is attributed to its role in promoting cellular cholesterol efflux playing a key role in reverse cholesterol transport.

Epidemiological studies have shown an inverse correlation between plasma concentrations of HDL cholesterol and cardiovascular risk [1]. The Framingham heart study which followed 5209 men and women for a period of 12 years reported that every 10 mg/dl increase in HDL cholesterol is associated with a significant decrease in the relative risk for coronary heart disease morbidity in 19% of men and 28% of women [2].

## 2. Composition of HDL

HDL cholesterol is a macromolecular complex of lipids and proteins and is highly heterogeneous in its physiochemical properties, metabolism, and biological activity [3]. Such heterogeneity is the result of differences in the relative contents of apolipoproteins and lipids in the HDL. Multiple subfractions of HDL can be identified in the plasma based on density, size, charge, and composition. On the basis of density, plasma HDL are divided into HDL2 (larger and less dense) and HDL3 (smaller and denser), while agarose gel electrophoresis further discriminates the basic HDL fraction (*α*-lipoproteins) and a small fraction, pre-*β*-HDL.

Plasma HDL are spherical or discoidal particles of highly hydrated density (1.063–1.21 g/mL) due to elevated protein content [4]. Discoidal HDL are small lipid-poor nascent particles primarily made up of apolipoprotein A-I (Apo A-I) embedded in a monolayer constituted of phospholipids and free cholesterol. Spherical HDL are larger mature particles additionally containing a hydrophobic core of cholesteryl esters and small amounts of triglycerides.

### 2.1. Proteins

HDL is the smallest lipoprotein with the highest density due to its high protein content. Apo A-I is the major protein component of HDL cholesterol making up 70% of its protein mass. Apo A-II comprises 15–20%, and the remaining protein mass comes from minor amphipathic proteins such as Apo C, Apo E, Apo D, Apo M, and Apo A-IV; enzymes such as paraoxonase (PON) 1, platelet-activating factor acetylhydrolase (PAF-AH), and glutathione peroxidase 1; lipid transfer proteins such as lecithin:cholesterol acyltransferase (LCAT) and cholesteryl ester transfer protein (CETP) [5].

### 2.2. Lipids

In addition to proteins, HDL cholesterol also contains lipids including free and esterified cholesterol; phospholipids including phosphatidylcholine, phosphatidylethanolamine, lysophosphatidylcholine, and plasmalogen; free fatty acids; mono-, di-, and triacylglycerols; sphingolipids such as ceramide, sphingomyelins, sphingosine-1-phosphate, lysosulphatide, and sphingosylphosphorylcholine [6].

## 3. Atheroprotective Functions of HDL

HDL cholesterol protects against atherosclerosis in multiple ways including reverse cholesterol transport and using antioxidant, anti-inflammatory, and antithrombotic mechanisms [7]. Although, our understanding of how HDL protects against coronary artery disease (CAD) is incomplete, we have evidence for three major atheroprotective mechanisms of HDL.

### 3.1. Reverse Cholesterol Transport

**Figure 1:**
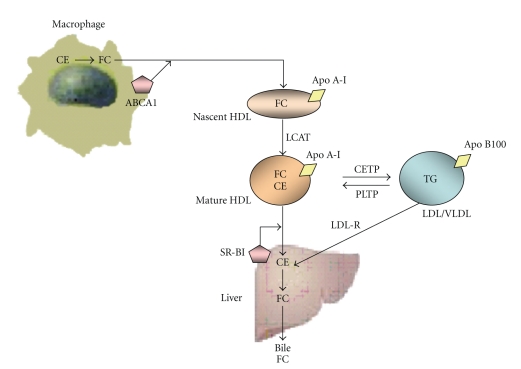
An overview of reverse cholesterol transport. HDL promotes the process of reverse cholesterol transport, whereby excess cholesterol present in the macrophage is effluxed to HDL and ultimately delivered to liver for excretion. HDL: high-density lipoprotein; CE: cholesterol ester; FC: free cholesterol; Apo A-I: apolipoprotein A-I; ABCA1: adenosine triphosphate-binding cassette transporter A1; LCAT: lecithin cholesterol acyltransferase; CETP: cholesterol ester transfer protein; PLTP: phospholipid transfer protein; LDL: low-density lipoprotein; LDL-R: low-density lipoprotein receptor; SR-BI: scavenger receptor class B-type I; VLDL: very low-density lipoprotein; TGL: triglycerides.

Reverse cholesterol transport is the primary mechanism by which HDL exerts its protective effect. It involves transport of cholesterol from peripheral blood cells, particularly macrophages, to the liver for excretion as bile acids and free cholesterol. This process is mediated by lipid transporter molecules such as ATP-binding cassette transporter A1 and G1 (ABCA1 and ABCG1) and scavenger receptor B-1 (SR-BI) [8]. HDL can deliver cholesterol to the liver through hepatic SR-BI, or alternatively cholesteryl esters within HDL are exchanged for triglycerides in low-density lipoprotein (LDL) or very low-density lipoprotein (VLDL) through CETP with subsequent hepatic uptake via the LDL receptor pathway. This potentially leads to recycling of cholesterol back into the artery wall, which is central to the atheroprotective role of HDL [8]. An overview of reverse cholesterol transport as shown in Figure 1. CETP is a critical modulator of HDL metabolism. CETP facilitates exchange of triglyceride for cholesteryl esters between triglyceride-rich lipoprotein particles (VLDL, IDL, and LDL) and HDL. This exchange results in increased cholesterol content of triglyceride-rich lipoprotein particles and cholesterol depletion of HDL particles. The small, cholesterol-deplete particles are often excreted in the urine. In metabolic diseases, such as type 2 diabetes and metabolic syndrome, elevated CETP activity results in increased cholesteryl ester (CE) transfer from HDL to triglyceride- (TG-) rich lipoproteins and in reciprocal TG transfer, producing TG-enriched HDL and decreasing HDL cholesterol levels [9]. Conversely, CETP deficiency reduces the exchange of TG and CE between HDL and TG-rich lipoproteins and elevates HDL cholesterol due to CE retention. As a consequence, increased CETP activity is thought to be proatherogenic in humans [10].

### 3.2. Inhibition of LDL Oxidation

In multicellular organisms, the major role of lipoproteins is extracellular transport of lipids. Apo A-I is a major LDL protein with a binding domain for LDL deposition in the extracellular matrix of many tissues, especially arteries susceptible to atherosclerosis. LDL binding to the subendothelial space causes cells to oxidize LDL lipids evoking the cells to secrete monocyte chemoattractant protein (MCP-1) and inducing an inflammatory response [11]. HDL in normal state abolishes the extracellular transport of lipids by preventing LDL oxidation, secretion of MCP-1, and the inflammatory response. Furthermore, HDL comprises a series of antioxidant enzymes which protect LDL from oxidation. Oxidized lipids are transferred to HDL from LDL and are hydrolyzed by HDL-associated PON1, LCAT, and PAF-AH enzymes [12, 13].

### 3.3. Anti-Inflammatory Properties

The anti-inflammatory activity of HDL is explained by its ability to selectively decrease endothelial cell adhesion molecules which facilitate the binding of mononuclear cells to the vessel wall and promote lesion development, thereby protecting against CAD [14]. HDL limits expression of cytokines such as tumor necrosis factor-*α* (TNF-*α*) and interleukin-1 that mediate upregulation of leukocyte-endothelial adhesion molecules. The ability of HDL to inhibit adhesion molecule expression could be mediated by apolipoproteins, not only by Apo A-I, but also by phospholipids, including sphingosine-1- phosphate and sphingosylphosphorylcholine [15, 16].

However, HDL has multiple additional endothelial and antithrombotic actions that may also afford cardiovascular protection which were discussed in detail in some recent reviews [17]. HDL modulates endothelial function, probably by stimulating endothelial nitric oxide (NO) production which is an atheroprotective signaling molecule. HDL stimulates NO production by upregulating endothelial NO synthase (eNOS) expression by maintaining the lipid environment in caveolae, where eNOS is colocalized with partner signaling molecules and by stimulating eNOS as a result of kinase cascade activation by the high-affinity HDL receptor SR-BI. HDL also protects endothelial cells from apoptosis and promotes their growth and their migration via SR-BI-initiated signaling.

## 4. HDL May Not Be Protective: Dysfunctional HDL

HDL and cardiovascular disease show an inverse correlation [1]. However, recent studies indicate that higher HDL levels may not always be protective and can become dysfunctional losing their cardioprotective effects [18]. HDL particles can vary in size, density, composition, and functional properties influencing their association with atherosclerosis [8]. Further, emerging evidence suggests that HDL function is not always accurately predicted by HDL cholesterol levels.

HDL acts as an anti-inflammatory molecule in healthy individuals. However, in those with chronic illnesses such as diabetes that are characterized by systemic oxidative stress and inflammation, HDL may actually promote the inflammatory response (i.e., it may become proinflammatory). More than a decade ago, Lenten and colleagues [19] reported that during an acute-phase response in animals or humans following surgery, HDL properties changed to become proinflammatory. These observations have formed the basis for subsequent studies evaluating the proinflammatory properties of HDL. At basal state, functional HDL shows high levels of antioxidants, active antioxidant proteins, and antioxidant enzymes with anti-inflammatory activity. However, when antioxidant and anti-inflammatory functions of HDL are overwhelmed by pathological processes such as inflammation, HDL is converted into a dysfunctional, proinflammatory particle that cannot promote cholesterol efflux or prevent LDL oxidation [20]. This dysfunctional HDL shows decreased levels and activities of anti-inflammatory and antioxidant factors, such as Apo A-I and PON1. Dysfunctional HDL contains oxidized phospholipids and proinflammatory proteins, such as serum amyloid A (SAA) and ceruloplasmin. Evidence shows that many pathological processes associated with systemic inflammation including chronic heart disease, metabolic syndrome, chronic kidney disease, infections, and rheumatic diseases are characterized by the presence of dysfunctional or proinflammatory HDL [21].

An oxidative environment is produced when an acute-phase response occurs as a result of nonspecific immunity. Thus, HDL appears to be part of the innate immune system and can be either proinflammatory or anti-inflammatory depending on the presence or absence of an acute-phase response and systemic inflammation.

**Figure 2:**
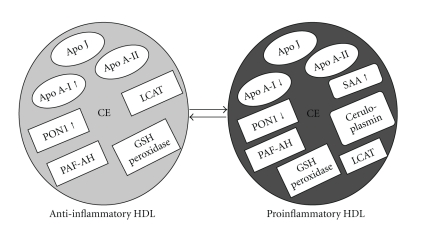
Model of bidirectional conversion of HDL from anti-inflammatory to proinflammatory. Normal anti-inflammatory HDLs are rich in apolipoproteins (ovals) and antioxidant enzymes (squares). After exposure to pro-oxidants, oxidized lipids, and proteases, proinflammatory HDLs have less lipoprotein and the major transporter apolipoprotein A-I are disabled by the addition of chlorine, nitrogen, and oxygen to protein moieties. PON1 cannot exert its antioxidant enzyme activity as Apo A-I can no longer stabilize it. In addition, pro-oxidant acute-phase proteins are added to the particle (serum amyloid A (SAA) and ceruloplasmin). “Apo A-I ↑”, “PON1 ↑” indicates that the number of respective molecules present in anti-inflammatory HDL is more when compared to that of proinflammatory HDL. Apo J: apolipoprotein J; CE: cholesterol ester; PON1: paraoxonase-1; GSH: glutathione; SAA: serum amyloid A; Apo A-I: apolipoprotein A-I; Apo-AII: apolipoprotein A-II; LCAT: lecithin cholesterol acyltransferase; PAF-AH: platelet-activating acyl hydrolase.

Change in HDL function parallels changes in HDL composition. Inflammation induces major changes in HDL levels and composition. Inflammatory cytokines such as TNF-*α* and interleukin-6 (IL-6) enhance expression levels of SAA and group IIA secretory phospholipase A2 (sPLA2-IIA) altering apolipoprotein content and levels [22, 23]. Myeloperoxidase (MPO) is a key inflammatory mediator of macrophages and other leukocytes, and systemic inflammation is thought to convert HDL to a dysfunctional form that loses its antiatherogenic effects. Modification of HDL composition by acute-phase response and oxidative stress is summarized in Figure 2. The pro-oxidant acute-phase reactants namely SAA and ceruloplasmin are associated with the formation of proinflammatory HDL along with Apo-j, also called clusterin [24]. The acute-phase HDLs are depleted in cholesterol esters but enriched in free cholesterol, triglycerides, and free fatty acids, but none of them can participate in reverse cholesterol transport or antioxidation [12, 25].

HDL undergoes pronounced structural and functional modifications in acute phase and inflammation. The major protein in HDL, Apo A-I, might be reduced because of decreased Apo A-I synthesis, accelerated HDL catabolism, and Apo A-I replacement by SAA. SAA is a pro-oxidant acute-phase reactant associated not only with disabling the anti-inflammatory role of HDL but also with creation of proinflammatory HDL [26, 27]. It is mainly of hepatic origin, and circulating levels can be induced to increase up to 1,000-fold in the presence of inflammation. Like C-reactive protein (CRP), elevated plasma levels of SAA represent an important, although weaker, cardiovascular risk factor. Recent studies suggest that Apo A-I oxidation by MPO results in the loss of HDL-mediated, antiapoptotic, and anti-inflammatory activities [28]. During acute and chronic inflammation, the content and functions of HDL can change drastically converting atheroprotective HDL to proatherogenic HDL.

HDL lipid composition might equally be altered during inflammation. Enrichment in TG with depletion of CE in the HDL core is the most frequent abnormality of HDL lipid composition and occurs in hypertriglyceridemic states associated with decreased activity of lipoprotein lipase, hepatic lipase, LCAT, or a combination of these. All these metabolic alterations are frequently observed in the acute phase and during inflammation [29]. In addition, HDL triglyceride content can also be increased in hypertriglyceridemia as a consequence of elevated CETP activity. CETP-mediated replacement of cholesteryl esters by triglycerides in the HDL core results in decreased plasma HDL cholesterol levels, which is another feature of the acute-phase response. Similar elevation in HDL-TG, decrease in HDL cholesterol, and increase in inflammatory markers are observed in the postprandial phase [30]. An elevated content of triglycerides might, therefore, represent a critical factor that lowers both HDL particle stability and plasma residence time. Acute-phase HDL also contains elevated levels of nonesterified fatty acids, lysophosphatidylcholines, and isoprostanes compared with normal HDL; in addition, CE levels are decreased [31].

Activities of the HDL-associated enzymes PON1, PAF-AH, and LCAT were indeed decreased in the acute-phase response. Van Lenten et al. noted that activities of PON1 and PAF-AH, that is, the anti-inflammatory properties of HDL, were restored upon resolution of the acute-phase response [19]. Further studies reported that PON1 and PAF-AH were partly responsible for the ability of HDL to inhibit LDL oxidation and the inflammatory response induced [32–34]. As part of the acute-phase response, activities of HDL-associated enzymes including PON1, PAF-AH, LCAT, CETP, and phospholipid transfer protein (PLTP) can be compromised, made dysfunctional or both [35].

Alterations occurring in HDL composition and metabolism due to inflammation are intimately associated with impaired biological activities. The cholesterol efflux capacity of HDL is considerably impaired during inflammation. Apo A-I, the major protein of HDL, plays an important role in the cellular cholesterol efflux, and the replacement of Apo A-I by SAA during inflammation can, therefore, have a significant impact on efflux. Enrichment of HDL with SAA results in increased HDL binding to macrophages, decreased cholesterol efflux from macrophages, and increased selective uptake of CE by macrophages [36, 37]. Importantly, SAA selectively impairs cholesterol efflux properties of small, dense HDL3 particles. Recently, McGillicuddy et al. provided evidence in humans and mice indicating that acute-phase HDL enriched in SAA induced by acute endotoxaemia have an impaired capacity to remove cholesterol from macrophages [38].

The antioxidative activities of HDL might equally become impaired in the presence of inflammation due to the replacement of Apo A-I by SAA and altered enzymatic activities [39]. Indeed, antioxidative deficiency of HDL relative to LDL oxidation by artery wall cells is observed in the acute phase, concomitant with decreases in the activity of PON1 and PAF-AH. All these mechanisms might limit the capacity of HDL to inactivate oxidized phospholipids, resulting in their elevated accumulation in LDL.

These altered HDLs are proinflammatory enhancing LDL oxidation and attracting monocytes to engulf the oxidized LDLs. Lipids in these HDLs are themselves oxidized. Van Lenten et al. were the first to report that during an acute-phase response, HDL loses its ability to inhibit LDL oxidation. They noted that HDL from normal rabbits and humans prevented LDL oxidation and LDL-induced MCP-1 production in cultures of human artery wall cells. In contrast, HDL isolated from the same source at the peak of an acute-phase response was less efficient in inhibiting LDL oxidation and increased MCP-1 production [19].

According to recent studies in patients with CAD, HDL is not only ineffective as an anti-inflammatory and antioxidant but is actually a proinflammatory and pro-oxidant promoting LDL oxidation. A study from Corsetti et al. suggested that raised HDL cholesterol levels and raised CRP levels may result in increased risk of cardiovascular disease. They also suggested that in patients with elevated levels of HDL and CRP, addition of CETP activity results in a higher CAD risk potentially explaining the negative findings from the torcetrapib studies [40].

The Thrombogenic Factors and Recurrent Coronary Events (THROMBO) postinfarction study by Corsetti et al. showed the same results in a subgroup of nondiabetic patients with high CRP levels who showed recurrent risk with increasing HDL cholesterol levels [41]. Extending these studies to a healthy population (Prevention of Renal and Vascular End-Stage Disease study) to determine whether primary coronary risk acted similarly identified a high-risk subgroup at high HDL cholesterol and CRP levels with presumptive evidence for large HDL particles. It also identified a second high-risk group with high CRP levels and low HDL levels as expected from many previous studies [42]. Subgroup patients had low levels of lipoprotein-associated phospholipase A2 (Lp-PLA2) and large HDL particles.

Rein et al. studied the roles of the metabolic syndrome, HDL cholesterol, and coronary atherosclerosis in subclinical inflammation and identified that the association of the metabolic syndrome with subclinical inflammation is driven by low HDL cholesterol [43].

## 5. Need for the Functional Tests

HDL cholesterol levels do not predict functionality and composition of HDL. The cholesteryl ester transfer protein inhibitor, torcetrapib that despite increasing HDL cholesterol concentrations failed the trial, has brought issues of HDL heterogeneity and function into sharp focus. In a controlled prospective trial on a combination of the HDL level-raising CETP inhibitor, torcetrapib, and statin by Barter et al., the HDL levels increased in 12 months in the torcetrapib/statin group, but the frequency of atherosclerotic events was significantly higher than the placebo plus statin group. However, the qualitative character of the increased HDLs was not measured in the study [44].

Plasma concentrations of HDL are insufficient to capture the functional variation in HDL particles along with the associated cardiovascular risk. These levels are also inadequate for assessing the potential therapeutic efficacy of novel HDL-targeted therapies. In light of recent developments, there is a growing need to identify other HDL-related subclasses and functions and biomarkers that better predict cardiovascular risk and can be used to assess the clinical benefits of novel HDL-targeted therapies.

Several ex vivo and in vitro assays have been developed to assess HDL's heterogeneity and its various functions. These tests are not yet commercially available but hold promise that we may be able to go beyond measuring the HDL cholesterol level and determine the functional characteristics of the patient's HDL. Substantial progress has been made in the development of robust and reproducible methods for assessment of HDL subclasses, and many of these assays are now commercially available. HDL heterogeneity was measured using various methods like analytical ultracentrifugation, gradient gel electrophoresis, 2-dimensional electrophoresis, and nuclear magnetic resonance spectroscopy. In contrast to the robust state of clinical chemistry regarding HDL subfractions, the laboratory assessment of HDL function remains in its infancy [45]. In vitro assays of HDL function have been developed by various research laboratories but are laborious, nonstandardized, and poorly validated with regard to human outcomes. There is an urgent need for producing meaningful and reproducible assays for various functions of HDL cholesterol like cholesterol efflux and reverse cholesterol transport, antioxidant, anti-inflammatory functions which are validated in large population.

## 6. Conclusions

Measuring HDL cholesterol levels may not predict functionality and anti-inflammatory properties of HDL. For this, we need to test the composition, functionality, and inflammatory properties of HDL. Though there are robust and reproducible methods for assessment of HDL heterogeneity, there are no widely available tests for measuring HDL functionality in clinical practice. In vitro assays for HDL function have been developed by various research labs but are nonstandardized and poorly validated. As such, there is a need for further research into development of standard methods to use these assays in large population-based studies and test whether they predict risk independent of HDL cholesterol concentrations.
